# New Insights Contributing to the Development of Effective Vaccines and Therapies to Reduce the Pathology Caused by hRSV

**DOI:** 10.3390/ijms18081753

**Published:** 2017-08-11

**Authors:** Nicolás M. S. Gálvez, Jorge A. Soto, Alexis M. Kalergis

**Affiliations:** 1 Millennium Institute of Immunology and Immunotherapy, Departamento de Genética Molecular y Microbiología, Facultad de Ciencias Biológicas, Pontificia Universidad Católica de Chile, Alameda 340, Santiago 8331150, Chile; nrgalvez@uc.cl (N.M.S.G.); jasoto6@uc.cl (J.A.S.); 2 Departamento de Endocrinología, Facultad de Medicina, Pontificia Universidad Católica de Chile, Santiago 8331150, Chile

**Keywords:** hRSV, vaccine, respiratory viruses, prophylaxis, BCG

## Abstract

Human Respiratory Syncytial Virus (hRSV) is one of the major causes of acute lower respiratory tract infections (ALRTI) worldwide, leading to significant levels of immunocompromisation as well as morbidity and mortality in infants. Its main target of infection is the ciliated epithelium of the lungs and the host immune responses elicited is ineffective at achieving viral clearance. It is thought that the lack of effective immunity against hRSV is due in part to the activity of several viral proteins that modulate the host immune response, enhancing a Th2-like pro-inflammatory state, with the secretion of cytokines that promote the infiltration of immune cells to the lungs, with consequent damage. Furthermore, the adaptive immunity triggered by hRSV infection is characterized by weak cytotoxic T cell responses and secretion of low affinity antibodies by B cells. These features of hRSV infection have meant that, to date, no effective and safe vaccines have been licensed. In this article, we will review in detail the information regarding hRSV characteristics, pathology, and host immune response, along with several prophylactic treatments and vaccine prototypes. We will also expose significant data regarding the newly developed BCG-based vaccine that promotes protective cellular and humoral response against hRSV infection, which is currently undergoing clinical evaluation.

## 1. General Features of Human Respiratory Syncytial Virus (hRSV)

Human Respiratory Syncytial Virus (hRSV), recently renamed as human orthopneumovirus [1], is an enveloped virus belonging to the *Pneumoviridae* family and the *Orthopneumovirus* genus [1]. This virus is considered the leading cause of acute lower respiratory tract infections (ALRTI) worldwide [2,3,4,5]. Although hRSV was first identified over 60 years ago, prior to that clinical cases without a clear medical report could be associated with the pathology induced by this virus [6,7,8]. The particular capacity of hRSV to induce the formation of syncytia on infected cells in culture was the reason of the initial naming [6,7]. Currently, hRSV is acknowledged as responsible for bronchiolitis and lower tract diseases affecting individuals of all range of ages with a varying degree of severity, with the most serious symptoms being shown by newborns, infants, the elderly and immunocompromised patients [4,9,10].

The immune response associated with the hRSV infection is characterized by an exacerbated inflammation in the airways, with impaired production of type III IFN [11,12]. Such a response resembles a Th2-polarized type of immunity that leads to the recruitment of inflammatory cells into the airways, particularly neutrophils and macrophages, and causes significant damage to the lung tissue [11,12]. Furthermore, since repetitive infections can take place throughout the life of an individual and even during the same outbreak season, it has been suggested that this virus promotes an inefficient, short lasting adaptive immunity [13].

Remarkably, extra-pulmonary manifestations associated with hRSV infection have been systematically reported, including the reference of cardiovascular complications such as arrhythmias and myocardial failures [14,15,16], liver complications leading to hepatitis in children [17,18], and central nervous system damage, leading to encephalopathies and impaired learning [19]. Consistently with this notion, viral RNA can be detected in both cardiovascular and central nervous system tissues [20,21]. Furthermore, among the long lasting sequelae of hRSV infection in children is the induction of asthma and chronic allergic inflammation in the airways as a result of severe bronchiolitis after exposure to hRSV [22].

Despite significant research efforts, the licensing of a vaccine that could be both safe and effective to protect against hRSV infection has been unsuccessful to date, most likely due to the incomplete comprehension of the immune mechanisms associated with this viral infection. One major representative of such ineffective efforts was the Formalin-Inactivated hRSV vaccine (FI-RSV), which actually led to enhanced disease in infants, causing a predisposition to an exacerbated immunopathology due to a strongly biased Th2-like immune response [23,24,25]. Currently, a few vaccine prototypes are being developed, with most of them aiming to diminish the Th2-like immune response observed in the hRSV infection [26,27,28,29,30]. Among those, our laboratory has generated a vaccine prototype consisting of a recombinant Mycobacterium bovis Bacillus Calmette–Guérin (BCG) that expresses the hRSV Nucleoprotein (rBCG-N), with promising results such as protective cellular and humoral responses [29,30].

In this article, we will discuss epidemiological data relative to hRSV throughout the years, the features of the virus genome and proteome, as well as the pathology that follows the exposure to this microbe. Further, we will go over the treatments developed to date, with special focus on a recombinant BCG vaccine prototype, detailing the cellular and humoral response that it can elicit in animal models.

## 2. hRSV Epidemiology

hRSV presents only one serotype, with two major antigenic groups, hRSV-A and hRSV-B. However the incidence of each antigenic group is highly variable in different locations around the world [31].

Currently, hRSV is considered one of the most significantly dangerous pathogenic agents by the World Health Organization (WHO) and the Center for Disease Control and Prevention (CDC), but there are no detailed epidemiological reports yet that could allow a rigorous characterization and updated information control [32,33].

Some of the epidemiological reports have suggested that in the year 2005, about 33.8 million children under the age of five could have been infected with hRSV, thus being responsible for almost 22% of the ALTRI worldwide [4]. Nonetheless, reports associated with the hRSV infection could be almost twice or even three times more frequent when data derived from children of one year old or younger are considered [4,34]. Furthermore, it was previously reported that about 66,000 and 199,000 children under the age of five that are hospitalized end up dying as a direct result of infection from this virus. Moreover, the most susceptible population to this phenomena are the children of under the age of 1 [4,34]. Significantly, the health burden associated to hospitalizations due to the hRSV-infection has been estimated to be about 394 million USD per year [35]. Moreover, although updated epidemiological studies remain very limited, projections suggest that the costs associated with hRSV-induced disease will increase over time.

## 3. Viral Genome and Protein Function

**Figure 1:**
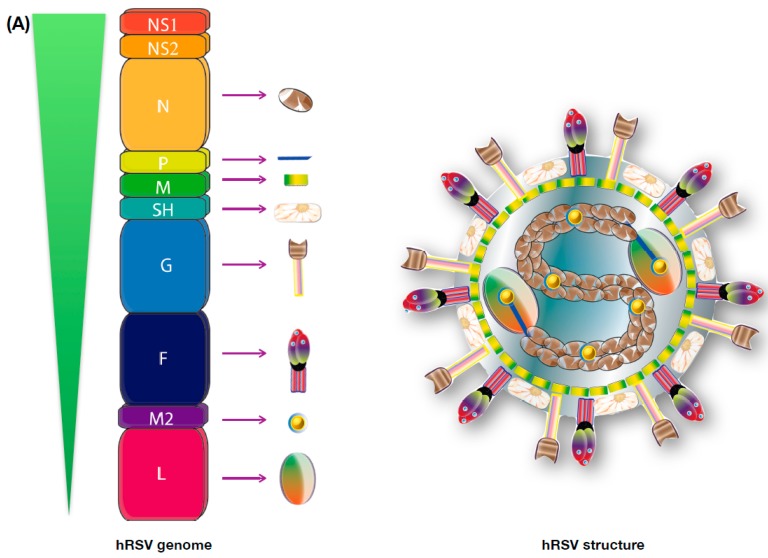

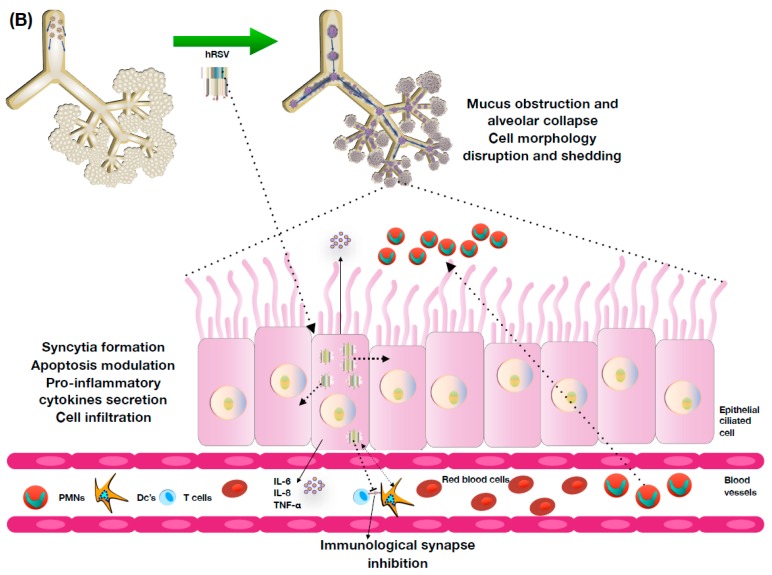
hRSV structure, pathology, and immune response.(**A**) The hRSV is an enveloped virus with a non-segmented, negative-sense, and single-stranded RNA genome. It has a genome of 15.2 kb consisting of 10 genes that codifies for 11 proteins; (**B**) The main target of infection of this virus are the epithelial ciliated cells. Its lung pathology is associated with mucus obstruction of the airways and its consequent alveolar sparing. The immune response associated to this pathogen is characterized by infiltration of PMNs to the lungs (eosinophil, neutrophils, monocytes); inflammation of the distal bronchial airways; a weak and unresponsive T cell immunity, most-likely due to the inhibition of the proper assembly of the Immunological synapse; and the secretion of many pro-inflammatory cytokines, such as, IL-6, IL8, TNF-a, among others.

hRSV is an enveloped virus with a non-segmented, negative-sense, and single-stranded RNA genome, recently renamed as “Human orthopneumovirus” and also re-classified to the *Pneumoviridae* family and the *Orthopneumovirus* genus [1,8,10,36]. Importantly, the closely related *Metapneumovirus* genus, in which the human Metapneumovirus (hMPV) is included, was also re-classified into the *Pneumoviridae* family [1,37]. The hRSV has a genome of 15.2 kb consisting of 10 genes that encode 11 proteins and that organize as follows 3′-NS1-NS2-N-P-M-SH-F-G-M2.1-M2.2-L-5′ (Figure 1A) [38]. The expression level of these proteins declines in direct correlation with their genome location [38,39]. NS1 and NS2 are the two non-structural proteins encoded by the virus genome and are the most abundantly expressed proteins during the first stages of replication [40,41,42]. Both of them are considered major hRSV virulence factors, while NS1 impairs IFN secretion-signalling, inhibits apoptosis of infected cells, and modulates the innate immune response [40,42], NS2 promotes direct cell rounding and shedding into the large airways of infected ciliated cells in the lungs [42,43,44,45]. Although these two proteins cannot be detected in virions, they are readily found inside infected cells [36,40,45]. Among the structural proteins, nucleocapsid proteins include the nucleoprotein, the phosphoprotein, and the large RNA-dependant RNA polymerase (N, P, and L, respectively). These three proteins are in direct contact and wrap the genomic viral RNA, with the phosphoprotein being able to generate complexes with the N protein and the large polymerase being responsible for the genomic replication [46]. The nucleoprotein has been also shown to locate at the membrane of infected cells, where it can modulate the function of immune cells [29,47,48]. The fusion disulfide-bonded glycoprotein, the large glycoprotein (F and G), and the small hydrophobic protein (SH) constitute the envelope proteins of hRSV. Both F and G are considered the two major antigenic determinants of hRSV [38,49,50]. The G protein is responsible for the attachment of the virus to new non-infected cells, while the F protein is in charge of the viral penetration and can induce syncytia formation [51]. The M2.1 and the M2.2 proteins are two different proteins encoded by the M2 gene, and are produced by the termination-dependent re-initiation mechanism [52]. They are considered nucleocapsid-associated proteins and are responsible for the assembly of the nucleocapsid and the modulation of the synthesis of the genome/anti-genome, respectively [36,52]. Finally, the matrix protein (M) is a non-glycosylated protein found just beneath the envelope and is the major lead in the assembly of the viral particle [38,53].

The main target of infection of this virus are the epithelial ciliated cells, although it can also infect some other cell types, such as basal cells, macrophages and dendritic cells [54,55]. In epithelial cells, the hRSV infection causes reduced cilia motility, altered apoptosis, and cell morphology disruption [42,43,45]. The infection cycle of the virus begins with the attachment to the host cells, through the G protein, and the subsequent entry process, in which the F protein is recognized by the nucleolin receptors located at the membrane of lung epithelial cells [51,56]. Upon entry of the virus, the envelope fuses with the cell plasma membrane, tagging the infected cells with several copies of the F, G, and SH proteins. Once in the cytoplasm, the viral nucleocapsid is released to initiate the transcription and replication of the proteome and genome of the virus [38,57]. Since hRSV is a negative stranded RNA virus, the synthesis of the polycistronic ssRNA positive-sense antigenome, which will be used as a template for the new negative-sense genome, is the first step of this process [36,49,50].

Both the F and, especially, the G protein are highly glycosylated, exhibiting 3 glycosylation sites for the F protein and about 40 for the G protein, a characteristic that allows them to partially avoid the immune system [58,59,60,61]. However, the F protein possesses several neutralizing epitopes, making it a good target for the antibody recognition [58]. In this line, and considering that both F and G are the main antigens located on the envelope of the virus and also on the membrane of the infected cells, the secretion of antibodies against hRSV and the humoral response elucidated by the immune system in the infants, although protective, is not enough to promote an effective clearance of the virus, since it is associated with the production of IgG1 and IgG3 antibodies, isotypes that have been described as not optimal during viral infections [58,59,62,63,64,65]. Remarkably, and as mentioned above, the nucleoprotein has been recently called to spotlight due to its novel capacity to migrate to the membrane of infected cells [29,47,48]. Considering that hRSV has the capacity to infect dendritic cells (DCs), among others cells of the immune system, this is a major concern in the formation of an effective immunological synapse (IS) and the subsequent activation of T cells [54,55]. It was recently shown that during early stages of the hRSV infective cycle, there is a significant expression of the hRSV-N protein at the surface of infected epithelial cells and DCs [47,48]. It has been suggested that expression of N at the membrane of infected cells requires a trans-golgi and lysosomal compartment exportation, from the cytoplasm to the membrane of the infected cells, since inhibition of these mechanisms also dampens the presence of this protein on the surface [27,30,48]. Moreover, the use of supported lipid bilayers, loaded with peptide-MHC complexes, an in vitro model that mimics the surface of DCs, could determine that the presence of the hRSV N protein is enough to inhibit activation of CD4+ T cells [48]. This is a new feature for the N protein, which can work as a virulence factor and contribute to modulate T cell immunity as it was shown for the nucleocapsid of the measles virus [66,67].

## 4. hRSV Pathology and Host Immune Response

The pathology associated to the hRSV infection is quite similar to the one observed in several other respiratory ailments caused by viral infections [2,67,68,69]. Although it is considered that the primary infection produced by hRSV is meant to be always symptomatic, some reports have suggested the presence of the viral genome in nasopharyngeal aspirates of asymptomatic children [70,71,72,73]. Among the most common symptoms induced by the infection in non-immunocompromised patients fever, nasal congestion, rhinitis, and coughing are the typically listed [38,39,74]. Along these, a normal response and lung pathology is associated with mucus obstruction of the airways, the consequent alveolar sparing, and a later viral clearance of the airways [13,75].

The natural immunity elicited by this virus is weak and fails to prevent the frequent re-infections observed throughout the life of an individual [49,76,77]. However, such an immune response displays a somehow protective profile that enables a healthy immune system to overcome the disease most of the time [50,76,77]. Although an immunity of the lower respiratory tract can be achieved early in childhood, lack of immunity in the upper respiratory tract is considered the main cause of the symptoms shown by hRSV-infected patients [78,79]. On the other hand, symptoms displayed by immunocompromised patients are usually associated with LRTI, bronchiolitis, tracheobronchitis, dyspnea, and wheezing [78,79,80]. Furthermore, several host factors contribute to the severity of the symptoms shown by children during the illness caused by hRSV, such as prematurity, low birth weight, and congenital diseases including cardiovascular diseases or pulmonary alterations. These susceptibility factors can contribute significantly to the morbidity and mortality resulting from hRSV infection [76,80,81]. Remarkably, a strong association has been reported between hRSV infection and asthma [69,82]. Thus, infected young infants are more susceptible to developing asthma during adulthood and, in turn, asthmatic children are more susceptible to suffering a hRSV infection [82,83,84]. The most severe cases of hRSV infection, as stated above, usually induce an exacerbated Th2-like response, although some studies have suggested that protective immunity can also be triggered [85,86,87,88]. This Th2-like immune response is characterized by an infiltration of PMNs to the lungs (eosinophils, neutrophils, monocytes), inflammation of the distal bronchial airways, a weak T cell immunity and the secretion of several pro-inflammatory cytokines, including IL-6, IL8, TNF-a, CXCL2, and CXCL10, among others [20,85,89]. Furthermore, the secretion of these pro-inflammatory cytokines promotes the recruitment of macrophages and DCs to the infected tissues that can also be infected by the virus and will eventually enhance the inflammatory state [54,55,90]. All of these inflammatory processes impair the capacity of the immune system to clear hRSV and reduce the cytotoxic activity of immune cells, particularly affecting T cells and natural killer cells [47,48,91,92].

Upon infection of epithelial cells, TLRs, NLRs, and RLRs are the first line of defense against hRSV, as these receptors promote signaling cascades that will induce the activation of the inflamassome and the secretion of IL-1β, NF-κB, and type I IFN [93,94]. Furthermore, it has been reported that several of the hRSV proteins are able to modulate these signaling pathways and therefore enhance the Th2-like pro-inflammatory profile that will promote virus persistence and dissemination [13,75]. For instance, NS1 and NS2 proteins are able to interfere the secretion of the type I IFNs [40,42,44,45,95]. Particularly, the F-protein is responsible of the production of pro-inflammatory cytokines such as IL-1β and IL-6, through the stimulation of the NF-κB pathway [96,97].

In addition, it has been reported that hRSV is able to interfere with the proper activation of the T cells by DCs, a phenomenon that involves the inhibition of the assembly of an effective immunological synapse [47,48]. The lack of proper activation has been suggested to be in direct association with the ineffective cellular immunity observed during hRSV infection, as an effective antiviral immune response should be associated with the secretion of granzyme B, perforine, and INF-y by the CD4+ T cells and the proliferation of the specific repertoire of CD8+ T cells, with the consequent increased secretion of IL-2, all of them absent in the pathology naturally induced by the virus [97,98,99,100,101]. Other significant characteristics of the immune response associated with this virus is the lack of development of an effective humoral response, as the infection induces the secretion of anti-hRSV antibodies with an IgG1 isotype by the B cells, which has been proven to be ineffective against the infection [102,103]. These antibodie isotypes are associated with a Th2-like immune response, once again dampening the antiviral response required for the resolution of the disease.

Though, and as described so far, the main features of the hRSV infection are associated with pulmonary pathology, recent reports suggest that infection with this virus is associated with neurological symptoms [19,20,21,104]. About a 2% of the children hospitalized by bronchiolitis also suffer from apnea, lethargy, seizures, and encephalopathy [8,19,74]. Recent studies suggest that this is in direct correlation with the presence of the viral mRNA and its proteins along several regions of the central nervous system, such as the cortex of the brain [104]. Although the mechanisms associated with this phenomena are still to be elucidated, recent reports indicate that learning difficulties and erratic behavioral responses are seen in mice, even 30 days after the hRSV infection has been resolved [19,104].

As stated above, the hRSV infection has also shown to cause abnormal pulmonary function, wheezing and asthma in childhood [82,105]. Furthermore, severe hRSV bronchiolitis in infancy is considered to be a risk factor for the development of allergic asthma [3,84,85]. Asthma is a chronic allergic inflammatory condition of the airways that affects more than 300 million people worldwide. This pathology is characterized by the activation of mast cells and the recruitment of eosinophil and Th2 lymphocytes to the lungs. This pathology is accompanied by an exaggerated sensitivity of the airways to nonspecific stimuli, known as a hyper-responsiveness (AHR), resulting in a widespread airflow obstruction and breathing difficulties [22].

Studies suggest that viral infections promote an exacerbation of allergic diseases, with rhinovirus (RV) being the main viral agent in humans. However, other viruses—such as hRSV, hMPV, influenza, parainfluenza, and adenovirus—have also been associated with exacerbation of allergy-related pathologies [69,82].

Particularly, hRSV has been described as an inducer of the polarization of naïve T cell onto a Th2 and Th17 profile and a mildly-induced Th1 profile [20,106]. Asthma pathology is also known to induce a Th2 immune response, in which a decrease in the secretion of IFN-γ and an increase in the secretion of IL-4 is reported. During the inflammatory process of asthma, multiple cytokines are secreted by alveolar epithelial cells, such as IL-25 (IL-17E), TSLP, and IL-33, that can initiate and modulate a Th2-like immune response [89]. Consequently, the Th2 immune response is characterized by the secretion of IL-13, an effector-cytokine in allergic responses [85]. Since both pathologies, hRSV infection and asthma, induce a similar response, it has been suggested that hRSV promotes an exacerbation in the asthmatic pathology [86].

## 5. Prophylaxis and Vaccine Development

Currently, the most effective treatment against hRSV is the use of a monoclonal antibody that targets the F protein of this virus, as a prophylactic method. The Palliative Antiviral Humanized Monoclonal Antibody (palivizumab (Synagis^®^), Medimmune, Inc., Gaithersburg, MD, USA) is administered to high-risk infants mainly, though not exclusively, since it can significantly prevent the most severe symptoms of LRTI induced by hRSV infection [107]. It is effectively approved by the USA Food and Drug Administration (FDA) and it represents one of the first lines of defense worldwide in dealing with the burden of hRSV in the health-system. As stated above, palivizumab targets a region of the hRSV F protein that is highly conserved between both antigenic groups, so its effectiveness is ensured in that manner [36,107,108]. Its major issue is the price/convenience relationship, since several doses of this drug must be administrated in order to keep the immune protection effective, considering it is a passive transfer of immunity, so no memory response is achieved by its administration. Along these lines, the development of new therapies is still pending to prevent/treat the hRSV infection.

Attempts to generate antibodies that can target several specifics antigens of the hRSV, including improved ones for the F protein, have been tested and developed, notwithstanding that they sustain the same issues of being massively distributed seen for palivizumab, since their cost of production remains quite high and their effectiveness could be improved. Some examples of this monoclonal antibodies, other than palivizumab, are motivizumab, or MEDI-524 and MEDI-8897, both of them targeting the F protein, with enhanced neutralizing capacity and mab 131-2G, an antibody that targets the G protein and that has shown, in mice, a decrease in the pulmonary inflammation associated with the viral infection [107,109,110,111,112,113,114,115].

The development of vaccines for the *Mononegavirales* order, particularly for the hRSV and hMPV, as two of the most significant respiratory pathogens in this order, has been tested thoroughly over the years, in order to improve the prophylactics methods used nowadays. Among the most remarkable failed attempts of achieving a long-lasting, safe, and effective immunity against hRSV, the Formalin-Inactivated hRSV (FI-hRSV) vaccine stands out [63,116]. In 1969, this vaccine prototype, that consisted of the formalin inactivated virus and alum as adjuvant, was tested. Although in other viruses, the use of this kind of vaccine seems to be effective [117], when this was assessed in both, animal models and in humans, the results were an exacerbation of the disease symptoms when a posterior natural hRSV infection was suffered [63,84,116]. Later, some studies suggested that this exacerbation was associated with the promotion of an allergic-like immune response induced by the immunization, with significant immune complex deposition and activation of the complement system [23,116]. Moreover, some studies have suggested that the modification of the epitopes present in the viral proteins are the main cause of this damaging immune response, that has been characterized as an unbalanced polarization of a Th1/Th2 immune response, with a high infiltration of PMNs cells, driven by the increased secretion of pro-inflammatory cytokines [36,118]. However, despite all this information, the specific mechanisms that underlie the enhanced disease induced by the FI-hRSV have not been clearly elucidated yet. The negative impact that this vaccine generated has somewhat diminished, but not depleted, the efforts to develop several more vaccines against this virus.

As some studies have shown, the immune response induced by the FI-hRSV vaccination promotes an increase in the CD4+ T helper cells as compared to the mock group, and lower cytotoxic CD8+ T cells recruitment to the lungs in both groups. In addition, it was found that these CD4+ T helper cells produced an increase in the secretion of IL-13 and IFN-γ, promoting both a Th-1 and Th-2 immune response. Finally, this effect seen for the CD4+ T helper cells and the IL-13 and IL-4 cytokines secretion was tested in STAT6 KO mice, reporting a decrease in the exacerbated immune response when the animals were stimulated with the FI-hRSV, as compared to the wild type control group, suggesting that the STAT6 pathway is likely responsible of the Th-2 like immune response induced by this FI-hRSV vaccination [116]. Another study performed to understand the effect induced by the FI-hRSV vaccination showed an increase in the CD4+ T helper cells and a decrease of the Tregs in the lungs. Such an increase in the CD4+ T helper cells was correlated with a higher weight loss and an enhancement of disease symptoms, whose phenotype was restored when Tregs were administered, suggesting a key role of these cells on the pathology associated with the FI-hRSV vaccine [119].

The induction of the secretion of antibodies against hRSV is a fundamental mechanism to confer protection in the host, so other approaches of vaccine prototypes intended to achieve this kind of response. An assay using nanoparticles of the hRSV F protein was evaluated in this topic [28]. The focus intended in this experimental vaccine prototype design was to promote the expression of antibodies that were homologous to palivizumab, but with an enhanced effective response. Indeed, the effect of the vaccine prototype was tested in animals infected with hRSV A and B serotypes and compared to FI-hRSV-immunized or palivizumab-treated animals. The researchers found an increase in the secretion of IgG-antibodies when compared to the controls and, significantly, these antibodies exhibited an enhanced neutralizing capacity. Among the infection parameters, the animals displayed a decrease in the viral load in the lungs, suggesting that this vaccine prototype, based in nanoparticles, could be effective against both hRSV serotypes as compared with the current palivizumab therapy [28].

The development of subunit vaccines against hRSV has been one of the tested strategies. Subunit vaccines consist of a single antigen, sometimes even a small protein epitope, of the pathogen from which the protection is meant to be achieved, instead of the full inactivated or attenuated microorganism, in this way lessening the chances of generating an adverse reaction [81,120,121]. Previous studies focused on developing hRSV subunit vaccines initially proposed both the G and F proteins as potential candidates, since they are considered the major antigenic determinants of this virus. However, considering the higher degree of variability presented by the G protein in its sequence [38], researchers have taken the F protein as the main option for the development of these vaccines.

The hRSV F protein is characteristic for exhibiting two conformational states, pre-fusion (PrF) and post-fusion (PoF), that vary substantially in the exposition of the Ø epitope and that are dependent on proteolytic cleavage catalyzed by the enzyme furin in two specifics sites of the PrF F protein [120,122]. This Ø epitope is a region considered the most significant target for antibody recognition in this protein, hence being crucial in the development of the next generation of vaccines [58]. Studies have shown that the PrF conformation is able to increase the levels of neutralizing antibodies, when compared to the PoF conformation [120].

The features of the monomeric PrF F protein used in this study were able to induce the secretion of high levels of neutralizing antibodies against hRSV. This was in direct association with the capacity to recognize the pre-fusion Ø epitope antigen, which seemed to be an excellent vaccine prototype to prevent infection of hRSV [120]. However, the major limitation found in this work lies on the instability of the protein when traces of the enzyme trypsin were present, since this enzyme is capable of easily destabilizing the spatial conformation of the PrF protein, making it a very unstable target, as it can interfere with the proteolytic cleavage induced by furin. As a result, researchers have proposed to work on new strategies in order to give greater stability to this peptide [120].

There are other studies addressing the generation of a recombinant PrF F protein as a vaccine candidate. In cotton rats immunized with either the PrF and the PoF conformational state (adjuvanted with aluminum phosphate) there was an evident reduction of the viral loads in the lungs of rats infected with hRSV. However, viral loads were still detected in the upper respiratory tract portion of the animals immunized with the PoF, whereas no viral loads were detected in the PrF immunized rats. Moreover, sera with neutralizing capacities were obtained in a significant quantity from animals immunized with the PrF F protein, as compared to the PoF immunized mice [123].

Among the recent attempts to develop a vaccine against hRSV, a new prototype using a recombinant influenza virus capable of expressing a chimeric protein (Influenza A virus PR8/hRSV.HA-F) was developed, which conferred protection in mice challenged with hRSV [124]. The main feature of this chimeric protein was the use of the antigenic site II (F243-294) from the hRSV F protein that is known to induce neutralizing antibodies. The recombinant virus that expresses the chimeric protein Hemagglutinin-F (HA-F) was tested for intranasal immunization in mice and several controls such as the FI-hRSV or live hRSV-intramuscular immunization were evaluated. The research found that this vaccine prototype induced the secretion of significant levels of neutralizing-antibodies against the Influenza A virus PR8 HA and hRSV, as compared with the other groups. Also, these animals showed a decrease in the viral titer correlated with a decrease in the plate forming units (PFU) and a minor inflammation in the lungs. In addition, other parameters such as cellular recruitment, cytokines secretion, and plaque restriction assays corroborated the protective profile [124].

Although considering that the G protein of the hRSV show a higher degree of variability as compared to the F protein, the recombinant influenza virus vector has also been tested as a vaccine approach for this protein. This recombinant Influenza A virus expressing the chimeric Hemagglutinin-G protein, PR8/RSV.HA-G, exhibited a decrease in the pathogenicity of the viral infection upon inoculation in mice, maintaining the immunogenic response. Significantly, inoculation with this virus induced the secretion of IgG2a isotype antibodies, which shows enhanced neutralizing capacities against the hRSV, the reduction of viral load in the lungs upon hRSV infection, and the reduction in the infiltration of PMNs to the lungs [125].

Another of the targets chosen for the development of vaccines is the SH protein, the depletion of which has been the main approach taken so far for this protein. Some studies have used RSV∆SH in a first inoculation and a posterior infection with wild type hRSV. The results have shown that mice inoculated with RSV∆SH exhibited a lower weight loss and decreases in the recruitment of inflammatory cells, secretion of CXCR1, and viral load in the lung. It was also found that animals immunized with RSV∆SH showed an increase in the IL-1β secretion, prompting the viral clearance [126]. Likewise, several studies using the SH protein as a vaccine approach had been used against the bovine respiratory syncytial virus (bRSV) [127,128] showing promising results regarding a protective role in the bovine model, promoting an increase in the IL-1β secretion, similar to ones seen for hRSV.

To date, other subunit vaccine studies have been performed using the M2-1, N, and P proteins of hRSV as antigens, which have been expressed as recombinant constructs, inside of the measles virus as an expression vector (MVAIK) [129]. The assessment of the MVAIK/hRSV/M2-1 vaccine prototype has shown a reduced Th2-like immune response, and particularly high levels of IFN-γ and cytotoxic CD8^+^ T cells, which are associated with an efficient Th1-like immune response [129]. On the other hand, studies assessing the F and the G antigens of hRSV showed high levels of neutralizing antibodies, but low cell-mediated immune responses were detected. When the recruitment of PMN cells to the lung was assessed after a challenge with hRSV in these studies, it was determined that both the MVAIK/hRSV/M2-1 and MVAIK/hRSV/NP formulations were able to significantly decrease inflammation and cell infiltration to the lungs as compared with the MVAIK/hRSV/F formulation, in which some inflammatory cells were detected in the lungs. This suggests that the use of both subunit vaccines MVAIK/RSV/F with MVAIK/hRSV/M2-1 or MVAIK/hRSV/NP may be the best way to generate a vaccine capable of inducing a balanced Th1/Th2 immune response against hRSV [129].

The Virus Like-Particles (VLPs) are a new vaccine prototype that has been used against several pathogens [130,131,132,133]. Although the first approaches associated with the development of VLPs against hRSV were unsuccessful, the most recent attempts that have been tested have shown promising results. Nowadays, some options of hRSV-VLPs are mainly associated with use of the F and M proteins. One of the main reasons to choose the F-protein as one of the antigens in these VLPs is that it has been previously reported to activate the TLR4 pathway that induces the secretion of pro-inflammatory cytokines. On the other hand, the M-protein—the other significant antigen used in the VLPs—is associated with the viral assembly and the interaction with some components presents in the infected cells [134,135]. The results obtained from these studies showed that both the F-hRSV-VLPs and the M-hRSV-VLPs vaccines were able to induce a higher antibody secretion in immunized mice, when compared with the control groups, independent of the administration route (i.n or i.m) [136]. Also, they observed that 21 days post-immunization, the IgG2a/IgG1 ratio was significantly higher in regards to the controls and FI-RSV immunized mice. Additionally, the cytotoxic CD8+ T cells were also able to promote an antiviral immune response that was evaluated by the cytokine levels secreted, the cellular infiltration of the lungs, and the viral load in the animals. The data clearly suggested that both vaccines had a protective role against both serotypes of hRSV, promoting an antiviral Th1-like immune response in contrast to the FI-hRSV vaccine, which induces an inefficient and exacerbated Th2-like immune response with an allergic-like profile response [136].

Considering that the population most endangered by hRSV is newborns, maternal immunization during the pregnancy has been a significant edge of study, although just a few studies are published in this field and there is still controversy in this aspect [26,137,138,139,140,141,142]. It has been previously seen that the transfer of antibodies with neutralizing capacities against hRSV, generated due to an infection or by an immunization, is effective, both through the placenta and through the breast milk [137,138]. However, there is discrepancy in whether these antibodies are effective against the infection, since some studies suggest that they could be beneficial in protection, while others suggest that they actually can induce airway hypersensitivity and recurrent wheezing, failing to protect against the virus [72,140,141]. Moreover, studies have shown that the presence of high titers of anti-hRSV antibodies in infants were dampening in the capacity to produce self-made antibodies against the virus [141]. In this line, the parameters in which the infection or the immunization occurs, such as the pregnancy stage or the maturity of the immune system of the newborn, may be significant in the production of antibodies that effectively protect against hRSV.

## 6. The rBCG-N-hRSV Vaccine

Considering all the data regarding the misbalanced Th2-like response associated with the hRSV infection, recently a new vaccine prototype has been developed using a recombinant mycobacterium bovis BCG as a vector to express the nucleoprotein present in hRSV (rBCG-N). This bacteria has been widely used for almost a century to prevent Tuberculosis disease, with clear characterization of its safety, and it has been thoroughly characterized as an effective Th1 immune response inducer [143,144]. Due to this last point, it has previously been tested as a recombinant vector for the expression of proteins of several pathogens, with promising results in vivo. Under this rationale, the effectiveness of the rBCG-N-hRSV has been tested in mice with positive effects being reported so far [29,30,145]. Particularly, the results found were associated with a protective immune response, with a clear tendency to a Th1-like profile, when mice were immunized with the rBCG-N, as compared with the BCG-WT immunized mice and the control mice. It was reported that the rBCG-N immunization induced a significant increase in the amount activated T helper cells (CD4+/CD69+) and cytotoxic T cells (CD8+) that were also able to secrete high levels of IFN-γ+ and IL-2, when compared with the non-vaccinated animals. Also, a decrease in the weight loss, the PMN cell infiltration, and the viral load in the lungs were reported. Additionally, an adaptive immunity transfer assay to naïve mice was performed, with the transferring of either or both helper and cytotoxic T cells (CD4+ and CD8+), reporting a protector role when both T cell types were transferred together, with an increased activation of cytotoxic CD8+ T cells when the animals were challenged with the virus. Remarkably, through the use of RAG KO animals, the authors also determined that the production of IFN-γ was necessary in order to achieve the protective response observed in the immunization with the rBCG-N-hRSV [29,145].

The rBCG-N vaccine prototype is also able to induce an effective humoral immune response against the viral infection, as described posteriorly. This antibody response is associated with the secretion of IgG antibodies specifically targeted against hRSV and its N protein, which is dependent of a posterior viral challenge (Unpublished data). Remarkably, there is also a significant increase in the secretion of antibodies against several other proteins of the virus—such as the G and P proteins—after a challenge with hRSV, although these are not part of the original vectored-vaccine. This event was addressed as Linked Recognition, a phenomenon in which the production of antibodies against proteins different than the one initially presented as antigen to B cells is achieved. This takes place when two or more proteins have epitopes spatially close to each other in a manner that the B cell presents on its MHC-II one of those linked antigens to the T cell. As a result, the B cell receives signals from the T cell, becomes activated and reacts to the secondary antigen; the one not presented to the T cell, and therefore generates antibodies against that linked antigen [146,147]. The vaccine was also able to promote switching from an IgG1-isotype to an IgG2a-isotype post viral challenge, which is a more adequate response of the immune system to face a viral infection. The antibodies, induced by the immunization and the subsequent infection, were able to neutralize the virus in vitro, diminishing its capacity to infect cells. Finally, the passive transfer of these antibodies promoted an effective protector role when the transferred naïve mice were challenged with hRSV (Unpublished data).

**Figure 2:**
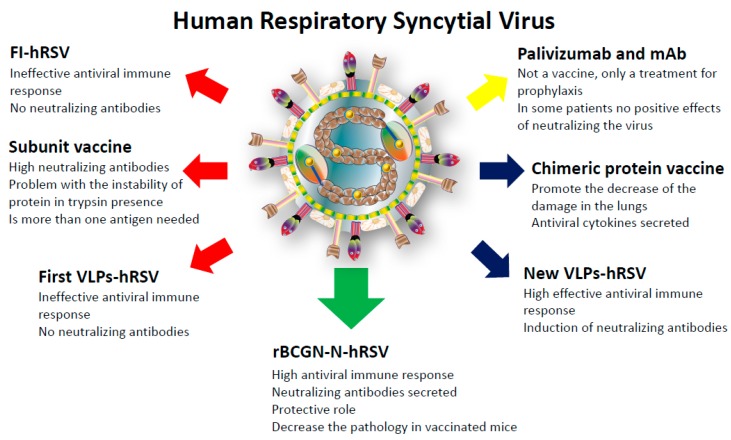
Vaccines prototype and prophylaxis methods used against hRSV. Several vaccine prototypes and prophylaxis methods have been tested against hRSV with a wide range of responses. The red arrows indicate failed vaccines attempts. The yellow arrow indicates only prophylactic methods. The blue arrows indicate potential candidates for an effective and safe vaccine, with testing still needed. The green arrow indicates the actual vaccine rBCG-N undergoing the Phase 1 clinical trial.

The information discussed above associated with the prophylactic methods, the vaccine prototypes, and the rBCG-N-hRSV vaccine are summarized in the Figure 2. Altogether, the data obtained so far from immunization with the rBCG-N vaccine suggests that it represents an effective and safe treatment to prevent the exacerbated symptoms induced by the hRSV infection. Significantly, this vaccine is now currently undergoing a Phase 1 clinical trial, in which its safety and dose-ranging are being tested and monitored in humans.

## Abbreviations

ALRTI	Acute lower respiratory tract infections
BCG	Mycobacterium bovis Bacillus Calmette-Guérin
DCs	Dendritic Cells
hMPV	Human Metapneumovirus
hRSV	Human Respiratory Syncytial Virus
IFN	Interferon
PMNs	Polymorphonuclear cells
rBCG-N	Recombinant Mycobacterium bovis Bacillus Calmette-Guérin
ssRNA	Single-stranded RNA
